# The Physiological Action of Urethane

**Published:** 1886-06

**Authors:** 


### The Physiological Action of Urethane.

Prof. Coze, of Nancy, sums up the physiological action of urethane, the new hypnotic, as follows:

1. Urethane has a marked hypnotic action, causes muscular relaxation, and, in large doses, anæsthesia.

2. It lowers the pulse and respiration, and reduces temperature.

3. It is slightly irritant, but may be administered subcutaneously.

4. It does not disturb nutrition, nor the fluids of the body.

5. It is, physiologically, an antidote for strychnine.

6. It should be tried in all cases of convulsions, particularly tetanus.

